# Stroke in Young Adults

**DOI:** 10.3390/jcm12154999

**Published:** 2023-07-29

**Authors:** Syed Bukhari, Shadi Yaghi, Zubair Bashir

**Affiliations:** 1 Temple University Hospital, Philadelphia, PA 19140, USA; 2 Department of Medicine, Brown University, Providence, RI 02912, USA; shadi_yaghi@brown.edu (S.Y.); zubair_bashir@brown.edu (Z.B.)

**Keywords:** stroke, mechanisms, dissection

## Abstract

Stroke in young adults is associated with significant morbidity, and its prevalence is rising in the United States. This is partly attributed to a rise in the prevalence of traditional risk factors including hypertension, hypercholesterolemia, obesity, diabetes mellitus, smoking and heart disease. In addition, there are non-modifiable risk factors comprising migraine, pregnancy and postpartum state, illicit drug use, oral contraceptives and hypercoagulable state. The mechanisms causing stroke in young adults are unique and include cervical dissection, cardioembolic phenomenon, vasculitis and vasculopathy, connective tissue disease, patent foramen ovale and cerebral venous thrombosis. The diagnosis of stroke in the young population can be challenging given its myriad clinical presentations. In this document, we provide an overview of the epidemiology of stroke in young adults, explore mechanisms that may explain increasing rates of stroke in this population, and provide a critical updated overview of the existing literature on the management and prevention of stroke in young adults.

## 1. Introduction

Cardiovascular diseases are the leading cause of global mortality and are associated with reduced quality of life [1,2]. Stroke is the second leading cause of death globally, and is recognized as the leading etiology of long-term physical and cognitive disability in adults [3,4]. In the United States (US), stroke is the fifth leading cause of death, and the economic burden of treatment and post-stroke care is substantial [5,6].

Traditionally perceived as a disease of older age, the increasing burden of stroke as a public health issue in younger individuals is alarming, with stroke in individuals <50 years of age accounting for ~10% of all strokes [7]. In the US, the average age at stroke onset is decreasing, and stroke incidence and hospitalization rates are rising among young people [8]. Although mortality among young patients with stroke is lower than in the elderly, it is significantly higher than in the age-adjusted general population [9]. Amongst the survivors, many deal with psychosocial consequences as a substantial proportion cope with sequelae including permanent cognitive deficits, epilepsy, and chronic debilitating fatigue with poor functional outcomes [10,11,12].

In this review article, we provide a critical updated overview of the existing literature on the epidemiology, risk factors, mechanisms, management and prevention of stroke in young adults.

## 2. Epidemiology

The prevalence of stroke in young adults, defined as age < 50 years, is estimated to be about 10–14% of all strokes [13,14]. Unlike stroke in older adults, the incidence of ischemic stroke among young adults is rising globally. In the US, the incidence of stroke for adults aged 20–44 has increased from 17 per 100,000 US adults in 1993 to 28 per 100,000 US adults in 2015 [15]. In a nationwide cohort of >15,000 patients from Netherlands, the incidence of any stroke in young adults increased from 14.0/100,000 person years in 1998 to 17.2/100,000 person years in 2010 (*p* < 0.001), driven by an increase in those aged over 35 years and ischemic stroke incidence (46%) [16]. A French study that examined >4500 patients over the course of 27 years concluded that for adults <55 years old, the incidence for ischemic strokes increased from 11.6 per 100,000 adults in 1985–1993 to 20.2 per 100,000 adults in 2003–2011 [17]. Alarmingly high trends have been noted in the incidence of stroke in young adults in low- and middle-income countries. A population-based study from India estimated that in the period of 2003–2005, the average annual incidence of stroke was only 4 per 100,000 people in patients <40 years, while it was exceptionally higher, at 41 per 100,000, for the 40–44 age group [18].

Young women are at a disproportionately increased risk of ischemic strokes compared with their male counterparts. A recent meta-analysis of 19 studies that reported on sex-specific stroke incidence among young adults found that there were 44% more women ≤35 years old with ischemic strokes than men (incidence rate ratio, 1.44 [1.18–1.76], I2 = 82%) [19]. This gap seemed to narrow in young adults 35 to 45 years old, as there was no significant difference in stroke incidence noted in this age group (incidence rate ratio, 1.08 [0.85–1.38], I2 = 95%) [19]. The prevalence of atherosclerotic disease is lower in premenopausal women and rises in postmenopausal women, and therefore the role of nonatherosclerotic and nontraditional risk factors in young women may be more important in ischemic stroke [20]. A better understanding of these sex differences is important to be able to implement strategies to prevent and treat strokes in this age group more effectively.

The incidence of ischemic strokes in young adults has been reported to be higher among Black and Hispanic individuals compared with white individuals. A population-based incidence study that examined all cases of first stroke in Northern Manhattan from 1993 to 1997 found that stroke in the young was greatest for Black (relative risk, RR 2.4; 95% CI, 0.8–6.7) and Hispanic people (RR 2.5; 95% CI, 1.1 to 5.8) compared with white people [21]. A Baltimore-based study showed that Black people had higher rates of both ischemic and hemorrhagic (intracerebral hemorrhage) strokes than white people [22]. The cerebral infarction rates per 100,000 were 22.8 for Black males, 10.3 for white males, 20.7 for Black females and 10.8 for white females. The intracerebral hemorrhage rates per 100,000 were 14.2 for Black males, 4.6 for white males, 4.8 for Black females and 1.5 for white females. While studies have shown no racial disparity in access to care as demonstrated by the equal administration of tPA and mechanical thrombectomy and non-significant differences in length of stay, the early poststroke functional outcomes remained worse in Black and Hispanic patients [23]. Black patients also have the highest 30-day poststroke mortality [24,25].

## 3. Risk Factors

**Figure 1:**
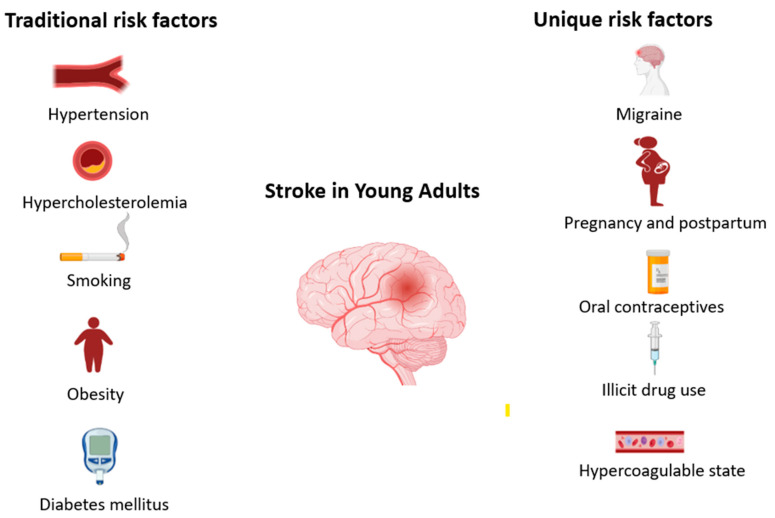
Risk factors for stroke in young adults.

The risk factors for stroke in young adults are largely similar to those for stroke in the general population, which are traditional factors including hypertension, hypercholesterolemia, obesity, diabetes mellitus, smoking and heart disease. However, there are additional factors that are unique to the young population and further potentiate stroke risk, including migraine, oral contraceptive use, pregnancy and post-partum state, patent foramen ovale and recreational drug use (Figure 1).

### 3.1. Traditional Modifiable Risk Factors

The rise in the incidence of stroke in young adults has been accompanied by an increase in modifiable vascular risk factors. In fact, the prevalence of having multiple traditional stroke risk factors among young adults with ischemic stroke has doubled over the decade from 2003–2004 through 2011–2012 [26]. Atherosclerotic disease accounts for about one third of ischemic strokes in young adults aged 15 to 45 years [26,27]. Notably, both small- and large-vessel disease have been identified as an increasing cause of ischemic stroke beginning at age 30 years [28]. Similarly, hypertension, hypercholesterolemia, obesity, diabetes mellitus and smoking are on the rise, contributing to increased stroke risk. 

### 3.2. Migraine

Migraine has been associated with both ischemic and hemorrhagic strokes in young adults [29]. In a meta-analysis that investigated the association between migraine and cardiovascular disease including stroke, migraine was associated with a twofold increased risk of ischemic stroke (RR 1.73; 95% CI, 1.31 to 2.29), and the risk was significantly higher among people who had migraine with aura (2.16, 1.53 to 3.03) compared with people who had migraine without aura (1.23, 0.90 to 1.69; *p*= 0.02) [29]. The relative risk for women was increased (RR 2.08, 95% CI 1.13 to 3.84) but not for men (RR 1.37, 95% CI 0.89 to 2.11), and the risk was further magnified by age < 45 (RR 3.95, 95% CI 2.21 to 6.04), smoking (RR 9.09, 95% CI 4.22 to 19.34) and oral contraceptive use (RR 7.02, 95% CI 1.51 to 32.68). Migraine predisposes patients to endothelial dysfunction and increased platelet activation and aggregation, thereby triggering key pathogenetic mechanisms of atherosclerosis and potentiating cerebral ischemia.

### 3.3. Pregnancy and Post-Partum

Pregnancy and the postpartum period are associated with an increased risk of ischemic stroke and cerebral hemorrhage. The risk of stroke among pregnant and postpartum women is 3 times increased when compared with nonpregnant women of similar age [30]. Preeclampsia and stroke have numerous risk factors in common, including metabolic syndrome, obesity, hypercoagulable states, endothelial dysfunction and accentuated inflammatory response [31]. 

### 3.4. Illicit Drug Use

The use of illicit and recreational drugs is on the rise among young adults [32]. Illicit drug users have an increased risk of both hemorrhagic and ischemic stroke. In the Baltimore–Washington Cooperative Young Stroke Study that included 428 patients 18 to 44 years old with first stroke, the use of illicit drugs was found to be the fifth most common risk factor for ischemic stroke (9.4% of the study population) [33]. Cerebral vasospasm is an important mechanism of cocaine-induced stroke. The most frequent mechanism of stroke in heroin addicts is cardioembolism due to infective endocarditis.

### 3.5. Oral Contraceptives

The role of oral contraceptive pills (OCPs) in stroke in young adults is controversial. The earliest meta-analysis of 16 studies from 1960 to 1999 found an elevated risk of stroke in young women who used high-dose estrogen-containing OCPs (RR 2.75, 95% CI 2.24–3.38), while progestin-only OCPs did not increase stroke risk [34]. A Danish study comprising 1,626,158 women showed that while the absolute risk of thrombosis with the use of hormonal contraception was low, the risk was increased by a factor of 0.9 to 1.7 with oral contraceptives that included ethinyl estradiol at a dose of 20 μg and by a factor of 1.3 to 2.3 with those that included ethinyl estradiol at a dose of 30–40 μg [35]. OCPs are associated with increased procoagulant factors, including fibrinogen, prothrombin and factors VII and VIII, and a decrease in the levels of antithrombin and tissue factor pathway inhibitor. In addition, OCPs, through the induction of hypertension, can increase the risk of hemorrhagic strokes [36].

### 3.6. Hypercoagulable States

Hypercoagulable states are common in young patients with ischemic stroke. Antiphospholipid syndrome, an autoimmune disease that occurs 5 times more often in young women, is present in 10% of stroke patients <50 years of age [37]. In a large multicenter population-based case–control study, a significantly increased risk of ischemic stroke was found in association with this condition in women under 50 years of age (OR 43.1, 95% CI 12.2–152.0) [38]. Antiphospholipid syndrome occurs frequently among individuals with systemic lupus erythematosus, and several studies have shown that stroke risk is ~2 times higher among those with systemic lupus erythematosus, particularly at younger ages [39]. Sickle cell disease is another hypercoagulable condition, and about one quarter of patients with sickle cell disease have had a stroke by age 45 [40]. In addition, protein C and S deficiency (~6%), factor V Leiden (~6), antithrombin III deficiency (~7%) and prothrombin mutation (~4%) are other common prothrombotic states that increase the risk of stroke [37]. 

## 4. Mechanisms

**Figure 2:**
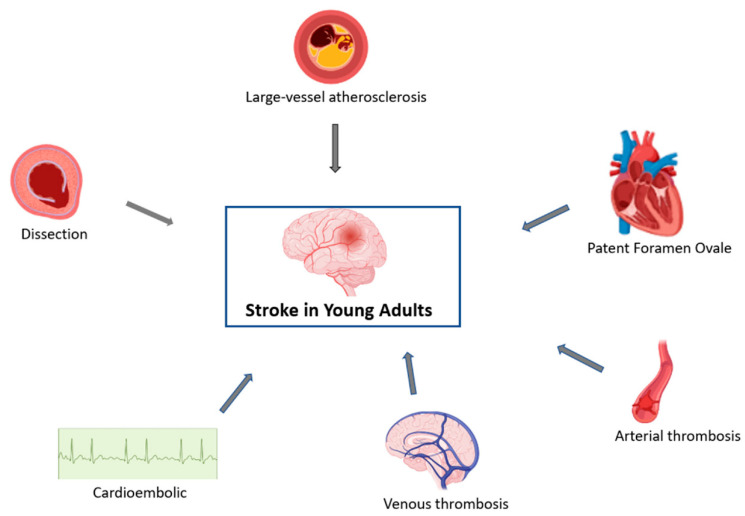
Mechanisms of stroke in young adults.

There are several mechanisms of stroke in young adults, including arterial, cardiac and venous (Figure 2). Extracranial arterial dissection is a significant contributor to the stroke burden in young adults, accounting for 10–25% of strokes in patients <45 years old, but only for 2% of all ischemic strokes [41]. The highest incidence of dissections lies in the fifth decade, and men and women are about equally affected [42]. While most arterial dissections occur spontaneously, some are associated with trauma and several genetic and connective tissue disorders such as fibromuscular dysplasia, Ehlers–Danlos syndrome and Marfan syndrome. Cervical artery dissections, in general, have a favorable prognosis because of the young age of the patients.

Arterial thrombosis is the predominant mechanism of stroke in young patients with an underlying prothrombotic state. There are several other rare but well-known vasculopathic conditions that can lead to ischemic and/or hemorrhagic strokes, including Fabry disease, Moyamoya disease, reversible cerebral vasoconstriction syndrome, giant cell arteritis, Takayasu arteritis, primary cerebral arteritis and radiation-induced arteritis. Large artery atherosclerosis and small-vessel diseases are a more common cause of stroke after the age of 35 years. In addition, celiac disease has recently been described as a hypercoagulable condition that might be associated with increased risk for stroke via several mechanisms, one including embolism and another due to cerebral sinovenous thrombosis [43].

With regard to cardiac causes, cardioembolism is an important mechanism of stroke in young adults. Atrial fibrillation/flutter detected on electrocardiogram and/or thrombus detected in any cardiac chamber would point towards this mechanism in a patient presenting with stroke. However, approximately 20% to 40% of these young patients with stroke are classified as having embolic strokes of undetermined source, because the underlying stroke etiology cannot be reliably identified despite recommended diagnostic workup. Patent foramen ovale is present in up to 45% of young patients with cryptogenic stroke, and has been independently associated with cryptogenic stroke in young adults (OR 3.70; 95% CI, 1.42 to 9.65; *p* = 0.008) [44].

In addition, human immunodeficiency virus infection has also been described as one of the mechanisms of stroke [45].

With regard to venous etiology, cerebral venous thrombosis accounts for 0.5–1.0% of unselected stroke admissions and is about three times more common in women than in men,, partly due to its association with pregnancy and postpartum as well as with the use of OCPs.

## 5. Clinical Suspicion and Management

The timely diagnosis of stroke is important to ensure prompt treatment and reduce morbidity and mortality. Although the typical presentation of ischemic stroke in young adults is similar to that in older adults, it is the atypical symptoms that can pose challenges for diagnosis [46]. Atypical symptoms such as neuropsychiatric symptoms, acute confusional state, chorea and dizziness can be difficult to diagnose as they are non-localizing. Other atypical symptoms include severe nausea, vomiting, cranial neuropathies, visual symptoms, pure sensory loss and monoparesis [47]. 

Clinical clues can help identify the etiology of stroke. Persistent headache or neckache after recent minor trauma or sudden neck movements, including chiropractic neck manipulation or vigorous exercise, are associated with cervical arterial dissection. Similarly, a painful Horner syndrome, or coexisting cranial neuropathies, should always raise suspicion for dissection, since all cranial nerves except the olfactory nerve may be affected by dissection. Computed tomographic or magnetic resonance angiography is the diagnostic modality of choice. The treatment is antiplatelet therapy. 

Persistent headache, particularly thunderclap headache, with or without focal neurological findings in young adults with hypercoagulable condition, pregnancy, puerperium or OCP use could point towards cerebral venous thrombosis. A history of arterial or venous thrombosis and history of pregnancy complications (e.g., three or more miscarriages, intrauterine death, premature birth due to high blood pressure, pre-eclampsia, HELLP syndrome or placenta failure) should lead to suspicion for antiphospholipid antibody syndrome. The treatment is antithrombotics.

The presence of atypical symptoms including earache, behavioral and cognitive symptoms, encephalopathy, seizures, fever, weight loss, rash, visual problems and other organ involvement (e.g., lungs, skin, joints) should raise suspicion for vasculitis as the underlying etiology for stroke. Laboratory workup including raised erythrocyte sedimentation rate or C-reactive protein and positive autoimmune/inflammatory workup is suggestive. CT with contrast and/or MRI often reveals multiple bilateral infarctions, at various stages, usually affecting different vascular territories and meningeal enhancement. Angiography shows focal or multifocal segmental narrowing of branches of cerebral arteries or occlusions. 

Recurrent thunderclap headaches lasting 1–3 h with or without focal neurological symptoms or seizures in a patient with uncontrolled hypertension should raise suspicion for reversible cerebral vasoconstriction syndrome. Angiography is the gold standard for diagnosis, revealing segmental narrowing of branches of cerebral arteries (string of beads). The treatment is blood pressure control.

For embolic stroke, electrocardiography, cardiac telemetry and transthoracic echocardiogram with bubble study are commonly performed to investigate abnormal heart rhythms, cardiac sources of emboli and patent foramen ovale. The treatment is anticoagulation for atrial fibrillation to mitigate the risk of thromboembolism.

In addition, polysubstance abuse is more prevalent in young adults, and hence comprehensive drug screening is crucial for a young patient presenting with stroke. 

## 6. Prevention

Ischemic stroke is a condition associated with up to four times higher mortality in young adults compared to healthy individuals. The socioeconomic and emotional burdens of stroke cannot be ignored, as up to one third of ischemic stroke patients remain unemployed for up to eight years following the episode [6]. Therefore, a comprehensive multidisciplinary approach is necessary to reduce the risks of primary and secondary events. Intensive blood pressure control, lifestyle modification, dietary changes and better glycemic control may be beneficial for young adults. Particular attention should be given to discouraging drug use and promoting smoking cessation. 

Active smoking is associated with a dose-dependent increase in the risk of strokes, and this risk remains high in young stroke survivors due to lower rates of quitting smoking compared to older adults [48]. A comprehensive program consisting of evidence-based individualized smoking cessation strategies, including effective pharmacotherapy, motivational counseling, cost-effective intervention provision and effective surveillance strategies, may benefit in terms of achieving higher rates of smoking cessation and abstinence.

Hypertension is one of the leading modifiable risk factors for stroke. A reduction in blood pressure provides the greatest benefit in reducing the risk of stroke compared to other clinical outcomes such as coronary events [49]. Studies have shown that individuals with blood pressure significantly higher than the goal may benefit most from blood pressure reduction [50]. Moreover, reducing either isolated systolic or diastolic blood pressure has also been found to provide benefits in terms of reducing the risk of stroke. Clinical guidelines recommend targeting blood pressure levels of ≤140/≤90 mmHg in non-diabetic patients and ≤130/≤80 mmHg in patients with diabetes mellitus or those who can tolerate it without severe adverse effects [51].

Diabetes mellitus is another modifiable risk factor that significantly increases the risk of stroke in young adults. Recent studies have found that young adult stroke patients with a history of diabetes mellitus are at higher risk of in-hospital mortality and long-term care facility admissions compared to their counterparts without diabetes [31]. We believe that glycemic control should be individualized and that young adults with a longer life expectancy and minimal cardiovascular disease burden may benefit from more stringent glycemic control if it can be achieved without recurrent hypoglycemia.

Obesity is also associated with a higher risk of stroke, and its prevalence is higher in diabetic patients [52]. Lifestyle modifications such as physical exercise for at least three to four sessions per week of moderate to vigorous intensity with each session lasting 40 min, salt restriction to <1.5 g/d and DASH and Mediterranean-type diets are recommended to reduce weight and achieve better glycemic control. Certain antidiabetics, including metformin, pioglitazone and semaglutide, have been shown to reduce the risk of stroke [53]. In conclusion, an individualized strategy that includes lifestyle modifications and pharmacotherapy can be adopted to achieve the best clinical outcomes for young adults at risk of stroke due to diabetes mellitus and obesity.

Treatment with statin therapy is recommended for primary prevention in patients estimated to have a high 10-year risk of cardiovascular events. A consensus is yet to be reached on targeting the low-density lipoprotein (LDL) levels in preventing stroke recurrence of atherosclerotic origin. Statins are the recommended first-line therapy, and these can be combined with ezetimibe for better lipid control and the achievement of LDL goals. Statins have the advantage of pleotropic effects in terms of stabilizing atherosclerotic plaques in addition to lowering the LDL levels [54]. While a detailed discussion of the mechanisms of different pharmacotherapies is beyond the scope of this review, we recommend a more aggressive strategy for controlling dyslipidemia in young stroke survivors to prevent the risk of future recurrent strokes.

Non-pregnant females of reproductive age who experience migraines with aura are at a higher risk for stroke, which is further elevated when these individuals use estrogen-based oral contraceptive pills (OCPs) and smoke [34]. It is crucial to develop an effective strategy that involves counseling on smoking cessation, alternative methods of birth control and effective management of migraine headaches in this population. Furthermore, ergots and triptans are not recommended for the treatment of migraines in individuals who have suffered an ischemic stroke. 

## 7. Prognosis

Older patients are at a higher risk for short-, intermediate- and long-term morbidity and mortality from stroke compared with younger patients <50 years old [55]. However, studies have also shown that older patients are less likely to be treated with guideline-recommended stroke therapies than younger patients [56]. Older patients also demonstrate more severe stroke deficits at presentation and have a higher in-hospital case mortality than younger patients [57,58]. Potential contributing factors for the excess mortality evident in older patients in these data include increased stroke severity, greater frequency of atrial fibrillation and higher prevalence of medical comorbidities [59].

## 8. Conclusions

The incidence of stroke in young adults is on the rise globally. While this could be attributed to increases in the traditional modifiable risk factors including hypertension, diabetes mellitus, obesity and hypercholesterolemia, other nontraditional factors also account for this. There are different stroke mimickers that can potentially delay the diagnosis of stroke. Timely diagnosis and prompt treatment are important. Prevention, both pharmacological and nonpharmacological, as well as primary and secondary, is the key.

## Data Availability

Not applicable.
